# Synthesis and structure of (*E*)-3,4,5-trihy­droxy-*N*′-(3,4,5-tri­meth­oxy­benzyl­idene)benzohydrazide monohydrate

**DOI:** 10.1107/S2056989025004001

**Published:** 2025-05-13

**Authors:** Mamadou Lo, Bineta Sene, Adji Fatou Fall Pouye, Arie van der Lee, Abdoulaye Gassama, Sébastien Richeter

**Affiliations:** a Département de Chimie, UFR des Sciences et Technologies, Laboratoire de Chimie Physique des Matériaux (LCPM), BP 523, Ziguinchor, Senegal; b Institut Européen des Membranes, Université de Montpellier, CNRS, ENSCM, 34095 Montpellier, France; c ICGM, Univ. Montpellier, CNRS, ENSCM, 34293 Montpellier, France; University of Neuchâtel, Switzerland

**Keywords:** crystal structure, gallic acid, acyl­hydrazone

## Abstract

In the structure of the title compound, C_17_H_18_N_2_O_7_·H_2_O, hydrogen bonds link three mol­ecules to the water mol­ecule. Additional hydrogen-bonding inter­actions connect two mol­ecules *via* the amide nitro­gen donor and two meth­oxy oxygen acceptors. This three-dimensional hydrogen-bond network has no particular directionality, and a slight disorder of two meth­oxy groups is observed. According to force-field calculations, weaker but more extended van der Waals inter­actions show a larger stabilization effect than the hydrogen-bond inter­actions, while hydrogen-bond analysis suggests that polymorphs of the compound may be found in the future.

## Abstract 2

In the structure of the title compound, C_17_H_18_N_2_O_7_·H_2_O, relatively strong bifurcated and simple hydrogen-bond inter­actions are present, together with extended van der Waals inter­actions. A hydrogen-bond coordination analysis suggests that polymorphs of the title structure may exist.

## 1. Introduction

Gallic acid is a secondary natural metabolite widely found in various fruits and vegetables (Zhou *et al.*, 2020 ▸; Hadidi *et al.*, 2024 ▸). Structurally, it is a low-mol­ecular-weight phenolic acid, identified as 3,4,5-tri­hydroxy­benzoic acid, and it is studied extensively for its remarkable anti­oxidant properties, which have applications in medicinal chemistry and various industrial sectors (Badhani *et al.*, 2015 ▸).

Acyl­hydrazones [*R*—C(=O)—NH—N=CH—*R*′] represent a class of organic compounds resulting from the combination of a hydrazone function and a carbonyl group. Synthetically, they are obtained through the condensation of a hydrazide, typically derived from a carb­oxy­lic acid or its ester, with an aldehyde or ketone (Oliveira *et al.*, 2022 ▸; Haranahalli *et al.*, 2019 ▸; Vlad *et al.*, 2024 ▸). This straightforward and efficient synthetic method accounts for its growing use in various fields of modern chemistry (Liu *et al.*, 2020 ▸). In materials chemistry, acyl­hydrazones are employed to develop promising materials such as reversible polymers (Ramimoghadam *et al.*, 2024 ▸) and tunable photoswitches (van Dijken *et al.*, 2015 ▸). Moreover, their structure exhibits keto–enol tautomeric equilibrium, which underlies their extensive use as multidentate ligands in coordination chemistry (Liu *et al.*, 2022 ▸). Additionally, the structural similarity between acyl­hydrazones and peptides explains their widespread application in medicinal chemistry. Numerous studies have shown that acyl­hydrazones exhibit a broad range of biological activities (Socea *et al.*, 2022 ▸; Kassab, 2023 ▸; Maia *et al.*, 2014 ▸).

In this context, acyl­hydrazones derived from gallic acid emerge as a particularly promising class of compounds, combining the anti­oxidant and metal-chelating properties of gallic acid with the versatile chemical behavior of acyl­hydrazones. This simple and efficient methodology allows access to a wide range of gallic acid-based acyl­hydrazones with tunable properties. As part of our research efforts, we successfully characterized the crystallographic structure of the title compound (TTMB·H_2_O), paving the way for further structural and biological investigations.
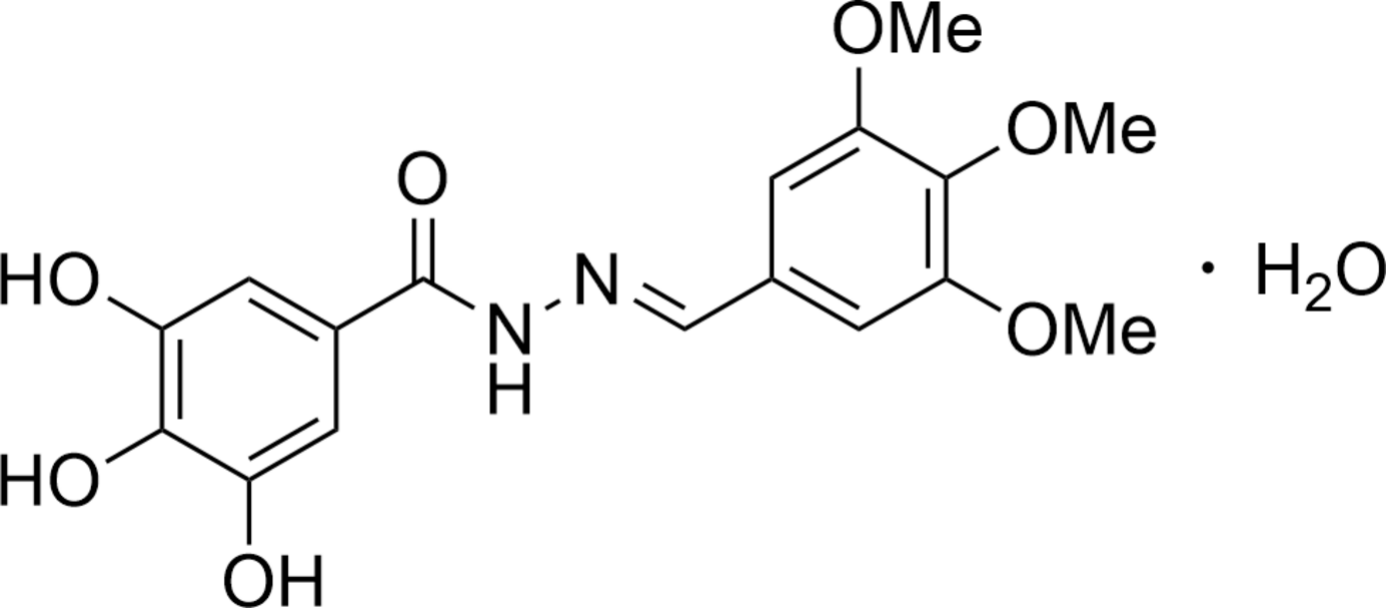


## 2. Structural commentary

**Figure 1:**
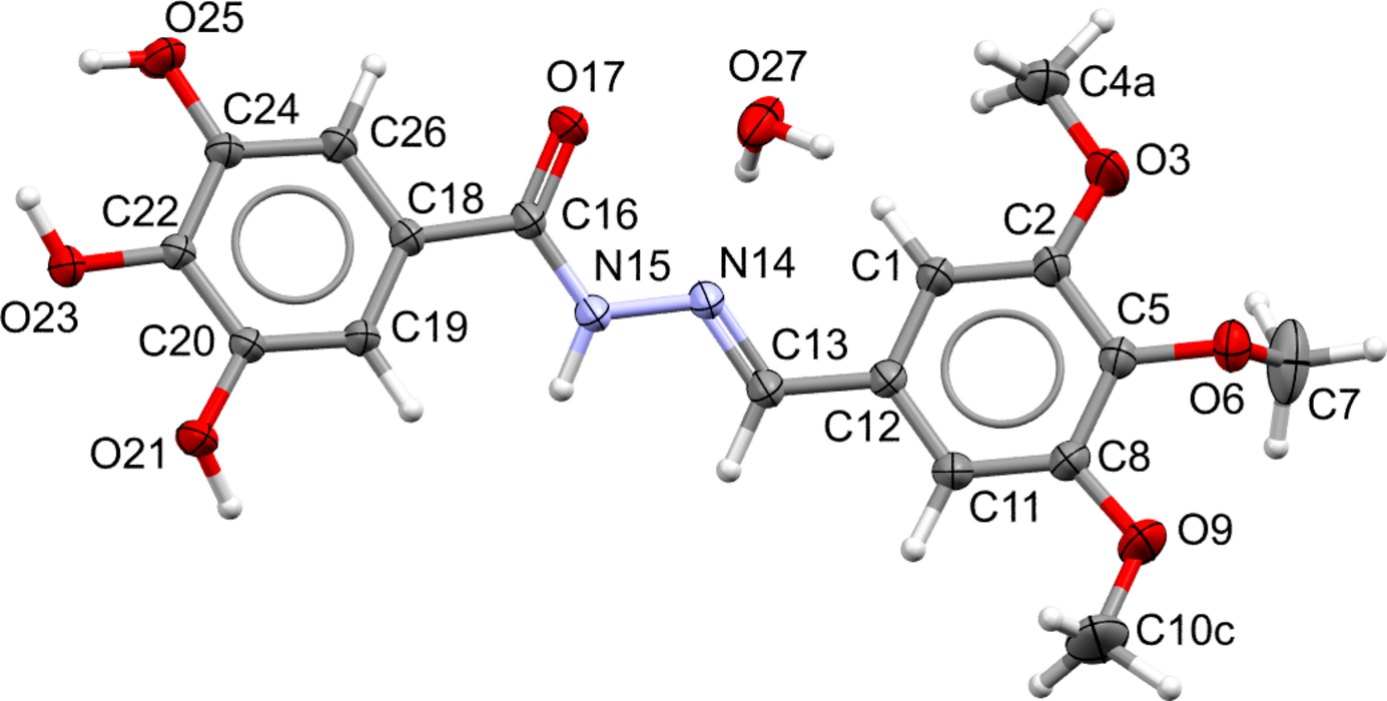
A view of the title structure showing the atom-labeling scheme. The atomic displacement ellipsoids are drawn at the 50% probability level. Only the major components of the disordered meth­oxy groups are shown. The isotropically refined hydrogen atoms have been drawn as fixed-sized spheres of 0.12 Å radius. The bond radius is 0.08 Å.

All bond lengths and angles are comparable to those of similar fragments present in the Cambridge Structural Database: a default *Mogul* check (Bruno *et al.*, 2004 ▸) gave no unusual features. The mol­ecular structure of the major disordered component of TTMB·H_2_O is shown in Fig. 1 ▸. The gallic acid phenyl ring and the trimeth­oxy phenyl ring are inclined slightly with respect to each other, subtending a dihedral angle of 10.56 (2)°. The dihedral angle between the central acyl­hydrazone motif and the trimeth­oxy phenyl ring is 14.01 (2)°, whereas that with the gallic acid phenyl ring is 12.64 (3)°. The meth­oxy group in the 3-position is in a close to perpendicular orientation with respect to the ring to which it is attached [C7—O6—C5—C2 torsion angle = −86.57 (9)°], whereas the disordered meth­oxy groups in the 2 and 4 positions are not far from coplanarity with the ring. The occupancy factors of the methyl groups of the 2,4 meth­oxy moieties are 0.54 (2)/0.46 (2) and 0.39 (3)/0.61 (3), respectively. The presence of the water mol­ecule in the crystal structure is probably due to traces of water in the ethanol used for the crystallization.

## 3. Supra­molecular features

**Figure 2:**
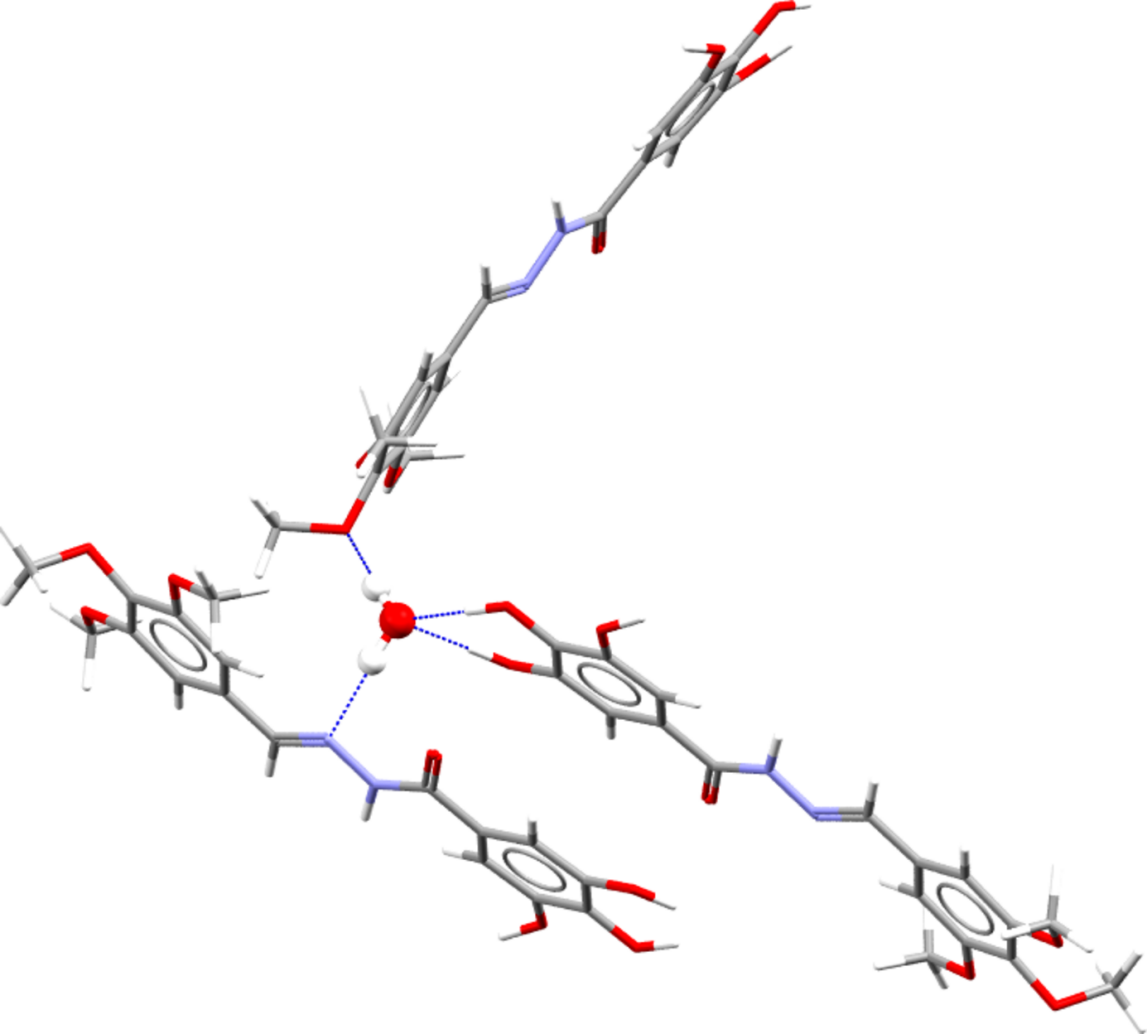
Hydrogen-bonded network between water and three TTMB moieties.

The most prominent supra­molecular feature in the structure of TTMB·H_2_O is the water mol­ecule that connects three TTMB moieties by short hydrogen bonds (Table 1 ▸, Fig. 2 ▸): donor–acceptor distances 2.8930 (8) (O27⋯N14), 2.7746 (8) (O27⋯O6), 2.7649 (8) (O25⋯O27), and 2.6892 Å (O23⋯O27). The latter two hydrogen bonds show a bifurcated bond involving two donor oxygen atoms of two neighboring hydroxyl groups of the same TTMB moiety, whereas the water oxygen acts as a single acceptor. An additional bifurcated hydrogen bond is found between two TTMB moieties, but involving the opposite configuration, *i.e.* two acceptor oxygen atoms and one donor nitro­gen. The two acceptor oxygen atoms belong to two neighboring meth­oxy groups and connect to an amide nitro­gen donor of a neighboring moiety. Such bifurcated hydrogen bonds are not rare and have been observed in α-helices, where it was shown that the inter­action energy of bifurcated hydrogen bonds is in general 50 to 60% smaller than those of a canonical single donor–single acceptor hydrogen bond (Feldblum & Arkin, 2024 ▸). A hydrogen-bond coordination analysis (Galek *et al.*, 2014 ▸) shows that the likelihood of an acyclic amide nitro­gen donor (N15) having a bifurcated hydrogen bond is very low (2.4%), compared to that of a – not observed – single hydrogen bond (86%). The probability of water oxygen atoms accepting two donors – as is observed in this structure – remains relatively low (29%) compared to the acceptance of only one donor (65%). Two of the three acceptor oxygen atoms of the meth­oxy groups show no inter­molecular inter­actions. The overall hydrogen-bond coordination capacity in TTMB·H_2_0 is only fulfilled at 56%, leaving the possibility open that one or more polymorphs may exist.

A full hydrogen-bond statistical analysis shows that the water oxygen (O27) to amide nitro­gen distance (N14) 2.8930 (8) Å, is unusually short, falling within the lower 5% qu­antile for 591 analogs. The same is true for the O27⋯O6 donor–acceptor pair distance [2.7746 (8) Å; 746 observations]. The N15—H151⋯O21 angle [121.6 (7)°] is also found to be unusually small, whereas the N15—H151⋯O23 angle falls within the expected range. This bifurcated hydrogen bond appears to be constituted of a strong and weak component.

**Figure 3:**
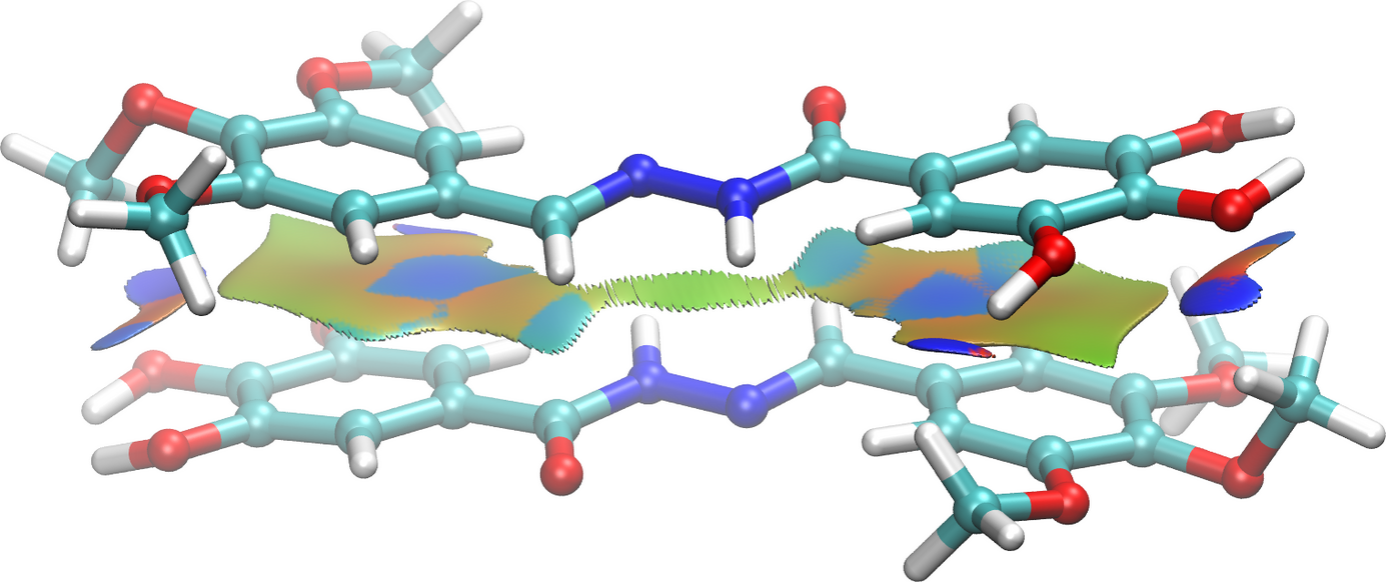
Non-covalent inter­action regions between two nearly parallel TTMB moieties. The reduced electron density gradient [*s*(ρ); see Johnson *et al.*, 2010 ▸] isosurface is drawn at the level of 0.3 atomic units and is color mapped based on the value of sin (λ)ρ: −0.1 (blue) < 0.0 (green) < 0.1 (red), where ρ is the electron density and λ the second electron density Hessian eigenvalue.

The hydrogen-bond network has no particular directionality and can thus be considered as three-dimensional. Although the hydrogen-bond network alone might seem essential for the stabilization of the structural architecture, there may be other inter­actions that play a role. There are no appreciable π–π inter­actions with centroid–centroid distances below 4.0 Å, and only one short C—H-centroid inter­action, C7—H73⋯*Cg*2(C12–C1–C2–C5–C8–C11), 2.608 (6) Å. The inter­action energies of different synthons that may contribute to the total lattice energy were calculated using the Momany force field, also called the CHARMm force field (Momany & Rone, 1992 ▸). In this force field, the inter­action energy of each synthon is decomposed in three different terms: an electrostatic, a hydrogen bond, and a van der Waals energy term. The most important individual synthon links two parallel TTMB mol­ecules with a slipped-parallel π–π stacking arrangement (Fig. 3 ▸). The energy decomposition shows that van der Waals inter­actions make up most of the total inter­action energy, which is confirmed by a pictorial representation of the inter­action zones calculated by the NCI index method (Fig. 3 ▸, Johnson *et al.*, 2010 ▸). The second most important synthon has two TTMB mol­ecules in a nearly perpendicular orientation for which the hydrogen-bond inter­actions are 40% more important than the van der Waals inter­actions. The synthons that link the water mol­ecule to the neighboring TTMB mol­ecules have the most important energy contribution, while the van der Waals energy is negligible. From the decomposition of the total lattice energy, it appears that – according to the Momany force field – approximately 46% of the stabilization is accounted for by van der Waals-type inter­actions, 31% by hydrogen-bond inter­actions and 23% by electrostatic inter­actions.

## 4. Database survey

A search of the Cambridge Structural Database (version 5.46 with November 2024 updates; Groom *et al.*, 2016 ▸) revealed 14 entries for gallic acid acyl­hydrazone derivatives, of which one contains a metal center (IDUVEK; Alhadi *et al.*, 2012 ▸). The hy­droxy­naphthalene derivative (LUYHUL; Shaikh *et al.*, 2020 ▸) was reported to be used as a nano-molar detection probe for the catalytic activity of La^3+^ ions *in vivo*. The structure that is most closely related to the title compound is the 2,4-di­meth­oxy­phenyl derivative (SOYCON; Alhadi *et al.*, 2009 ▸) but instead of a water solvate, it is an ethanol solvate. Among the fourteen related structures seven are water solvates, two are methanol solvates and one is an ethanol solvate, while the others have no solvent of crystallization.

## 5. Synthesis and crystallization

The title compound was synthesized in a round-bottom flask by dissolving 3,4,5-tri­hydroxy­benzohydrazide (1.1 mmol, 0.2 g) in absolute ethanol (2 mL). Subsequently, a solution of 3,4,5-tri­meth­oxy­benzaldehyde (1.0 mmol, 0.196 g) in absolute ethanol (2 mL) was added dropwise under continuous stirring, followed by the addition of a single drop of acetic acid as a catalyst. The reaction mixture was refluxed for approximately 3 h, leading to the formation of a white precipitate. The resulting solid was collected by filtration, washed thoroughly with cold ethanol, and air-dried. Single crystals suitable for X-ray diffraction analysis were obtained by slow evaporation of an ethanol solution of the title compound at room temperature over several days. Standard characterization data for TTMB are available in the literature (Peng *et al.*, 2022 ▸).

## 6. Refinement

Crystal data, data collection and structure refinement details are summarized in Table 2 ▸. An initial structure refinement was carried out using *OLEX2* (Dolomanov *et al.*, 2009 ▸) using spherical scattering factors for all atoms. The methyl groups of the two meth­oxy groups in the 2- and 4-positions were found to be slightly disordered over two different positions. The relative occupancies were refined while keeping the sum at 1.0 with soft restraints set on the oxygen-to-carbon distances, and similarity restraints on the atomic displacement parameters of each disordered group. The difference-Fourier map showed residual densities in the centers of almost all covalent bonds, so it was decided to perform the final refinement according to the Hirshfeld Atom Refinement (HAR) methodology using aspherical scattering factors as implemented in the *OLEX2* software package. The electron density was calculated from a Gaussian basis set single determinant SCF wave function (DFT-r2SCAN, cc-pVTZ basis set) with the *ORCA 6.0* package (Neese, 2012 ▸) and partitioning according to the NoSpherA2 methodology (Kleemiss *et al.*, 2021 ▸). The positions and isotropic atomic displacement parameters of the hydrogen atoms were freely refined. The four disordered parts were treated for the calculation of the wavefunctions according to the grouped parts method, giving four different structures (syntax 1-2;3,4 for parts 1/3, 1/4, 2/3, 2/4), recently described by Kleemiss *et al.* (2021 ▸).

## Supplementary Material

Crystal structure: contains datablock(s) I. DOI: 10.1107/S2056989025004001/tx2096sup1.cif

Structure factors: contains datablock(s) I. DOI: 10.1107/S2056989025004001/tx2096Isup2.hkl

Supporting information file. DOI: 10.1107/S2056989025004001/tx2096Isup3.cml

CCDC reference: 2449013

Additional supporting information:  crystallographic information; 3D view; checkCIF report

## Figures and Tables

**Table 1:** Hydrogen-bond geometry (Å, °)

*D*—H⋯*A*	*D*—H	H⋯*A*	*D*⋯*A*	*D*—H⋯*A*
O23—H231⋯O27^i^	0.941 (10)	1.748 (10)	2.6892 (7)	179.3 (9)
O21—H211⋯O17^ii^	0.955 (11)	1.790 (11)	2.7246 (7)	165.3 (10)
O25—H251⋯O27^i^	0.954 (11)	1.811 (11)	2.7649 (8)	177.5 (10)
N15—H151⋯O23^iii^	0.985 (9)	2.115 (9)	3.0491 (7)	157.7 (8)
N15—H151⋯O21^iii^	0.985 (9)	2.320 (9)	2.9572 (8)	121.6 (7)
O27—H271⋯O6^iv^	0.938 (11)	1.858 (12)	2.7746 (8)	165.1 (10)
O27—H272⋯N14	0.973 (12)	1.956 (12)	2.8930 (8)	161.0 (10)

Symmetry codes: (i) 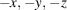; (ii) 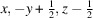; (iii) 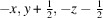; (iv) 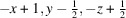.

**Table 2:** Experimental details

Crystal data	
Chemical formula	C_17_H_18_N_2_O_7_·H_2_O
*M*_r_	380.36
Crystal system, space group	Monoclinic, *P*2_1_/*c*
Temperature (K)	173
*a*, *b*, *c* (Å)	11.5409 (7), 10.9018 (6), 14.3799 (8)
β (°)	103.427 (2)
*V* (Å^3^)	1759.78 (17)
*Z*	4
Radiation type	Mo *K*α
μ (mm^−1^)	0.12
Crystal size (mm)	0.34 × 0.23 × 0.04

Data collection	
Diffractometer	Venture Photon-II
Absorption correction	Multi-scan (*SADABS*; Krause *et al.*, 2015 ▸)
*T*_min_, *T*_max_	0.95, 1.00
No. of measured, independent and observed [*I* ≥ 2u(*I*)] reflections	57780, 7401, 5802
*R*_int_	0.059
(sin θ/λ)_max_ (Å^−1^)	0.796

Refinement	
*R*[*F*^2^ > 2σ(*F*^2^)], *wR*(*F*^2^), *S*	0.035, 0.062, 1.08
No. of reflections	7401
No. of parameters	342
No. of restraints	16
H-atom treatment	Only H-atom coordinates refined
Δρ_max_, Δρ_min_ (e Å^−3^)	0.30, −0.30

Computer programs: *SUPERFLIP* (Palatinus & Chapuis, 2007 ▸), *OLEX2.refine* (Bourhis *et al.*, 2015 ▸), *Mercury* (Macrae *et al.*, 2020 ▸; *VMD* (Humphrey *et al.*, 1996 ▸) and *OLEX2* (Dolomanov *et al.*, 2009 ▸).

## supplementary crystallographic information

### (E)-3,4,5-Trihydroxy-N'-(3,4,5-trimethoxybenzylidene)benzohydrazide monohydrate Crystal data

**Table:**

C_17_H_18_N_2_O_7_·H_2_O	*F*(000) = 800.657
*M**_r_* = 380.36	*D*_x_ = 1.436 Mg m^−^^3^
Monoclinic, *P*2_1_/*c*	Mo *K*α radiation, λ = 0.71073 Å
*a* = 11.5409 (7) Å	Cell parameters from 9902 reflections
*b* = 10.9018 (6) Å	θ = 2.4–34.3°
*c* = 14.3799 (8) Å	µ = 0.12 mm^−^^1^
β = 103.427 (2)°	*T* = 173 K
*V* = 1759.78 (17) Å^3^	Prism, colourless
*Z* = 4	0.34 × 0.23 × 0.04 mm

### (E)-3,4,5-Trihydroxy-N'-(3,4,5-trimethoxybenzylidene)benzohydrazide monohydrate Data collection

**Table:**

Venture Photon-II diffractometer	5802 reflections with *I*≥ 2u(*I*)
φ and ω scans	*R*_int_ = 0.059
Absorption correction: multi-scan (SADABS; Krause *et al.*, 2015)	θ_max_ = 34.5°, θ_min_ = 2.4°
*T*_min_ = 0.95, *T*_max_ = 1.00	*h* = −18→18
57780 measured reflections	*k* = −17→17
7401 independent reflections	*l* = −22→22

### (E)-3,4,5-Trihydroxy-N'-(3,4,5-trimethoxybenzylidene)benzohydrazide monohydrate Refinement

**Table:**

Refinement on *F*^2^	28 constraints
Least-squares matrix: full	Primary atom site location: iterative
*R*[*F*^2^ > 2σ(*F*^2^)] = 0.035	Hydrogen site location: difference Fourier map
*wR*(*F*^2^) = 0.062	Only H-atom coordinates refined
*S* = 1.08	*w* = 1/[σ^2^(*F*_o_^2^) + (0.0094*P*)^2^ + 0.2877*P*] where *P* = (*F*_o_^2^ + 2*F*_c_^2^)/3
7401 reflections	(Δ/σ)_max_ = 0.001
342 parameters	Δρ_max_ = 0.30 e Å^−^^3^
16 restraints	Δρ_min_ = −0.29 e Å^−^^3^

### (E)-3,4,5-Trihydroxy-N'-(3,4,5-trimethoxybenzylidene)benzohydrazide monohydrate Fractional atomic coordinates and isotropic or equivalent isotropic displacement parameters (Å^2^)

**Table:**

	*x*	*y*	*z*	*U*_iso_*/*U*_eq_	Occ. (<1)
O23	−0.16740 (5)	−0.10296 (4)	−0.27135 (3)	0.01723 (10)	
H231	−0.2026 (9)	−0.1681 (9)	−0.2449 (7)	0.02584 (14)*	
O21	−0.00615 (5)	0.04187 (5)	−0.31462 (3)	0.02050 (11)	
H211	0.0150 (10)	0.1127 (10)	−0.3460 (7)	0.03076 (16)*	
O17	0.01977 (5)	0.26329 (5)	0.07329 (3)	0.01894 (10)	
O3	0.31182 (6)	0.71678 (6)	0.31595 (4)	0.03290 (15)	
O6	0.49176 (4)	0.83971 (5)	0.27220 (4)	0.01874 (10)	
O9	0.53534 (5)	0.82814 (6)	0.09931 (4)	0.03169 (14)	
O25	−0.23283 (5)	−0.07336 (5)	−0.09043 (4)	0.02225 (11)	
H251	−0.2469 (10)	−0.1477 (10)	−0.1263 (8)	0.03337 (17)*	
N15	0.10103 (5)	0.35562 (5)	−0.03782 (4)	0.01652 (11)	
H151	0.0992 (8)	0.3707 (8)	−0.1057 (6)	0.01982 (13)*	
N14	0.16027 (5)	0.43628 (5)	0.02928 (4)	0.01534 (10)	
C22	−0.11988 (6)	−0.01524 (6)	−0.20606 (4)	0.01278 (11)	
C20	−0.03649 (6)	0.06508 (6)	−0.23039 (4)	0.01397 (11)	
C19	0.01409 (6)	0.15878 (6)	−0.16865 (5)	0.01610 (12)	
H191	0.0802 (7)	0.2141 (8)	−0.1889 (6)	0.01932 (14)*	
C18	−0.01895 (6)	0.17342 (6)	−0.08161 (4)	0.01353 (11)	
C16	0.03388 (6)	0.26711 (6)	−0.00924 (4)	0.01391 (11)	
C13	0.21854 (6)	0.52293 (6)	0.00037 (5)	0.01643 (12)	
H131	0.2215 (7)	0.5313 (7)	−0.0733 (6)	0.01972 (14)*	
C12	0.28525 (6)	0.61051 (6)	0.06973 (4)	0.01459 (11)	
C1	0.25992 (6)	0.62084 (6)	0.15975 (5)	0.01662 (12)	
H11	0.1882 (7)	0.5690 (8)	0.1756 (6)	0.01994 (15)*	
C2	0.32763 (6)	0.69985 (6)	0.22673 (5)	0.01729 (12)	
C5	0.42032 (6)	0.76765 (6)	0.20393 (5)	0.01525 (12)	
C7	0.44587 (8)	0.96035 (8)	0.27896 (8)	0.0344 (2)	
H71	0.4434 (10)	1.0070 (10)	0.2129 (8)	0.0517 (3)*	
H72	0.3597 (10)	0.9547 (10)	0.2945 (8)	0.0517 (3)*	
H73	0.5075 (10)	1.0059 (10)	0.3340 (8)	0.0517 (3)*	
C8	0.44380 (6)	0.75848 (6)	0.11305 (5)	0.01781 (13)	
C11	0.37541 (6)	0.67979 (6)	0.04516 (5)	0.01825 (13)	
H111	0.3905 (7)	0.6718 (8)	−0.0256 (6)	0.02190 (15)*	
C26	−0.10082 (6)	0.09328 (6)	−0.05723 (5)	0.01540 (12)	
H261	−0.1239 (7)	0.1025 (8)	0.0107 (6)	0.01848 (14)*	
C24	−0.15188 (6)	−0.00032 (6)	−0.11857 (5)	0.01416 (11)	
O27	0.26649 (5)	0.28900 (5)	0.19444 (4)	0.02384 (11)	
H271	0.3461 (10)	0.3139 (10)	0.2147 (8)	0.03577 (17)*	
H272	0.2198 (10)	0.3454 (11)	0.1480 (9)	0.03577 (17)*	
C4a	0.2110 (5)	0.6593 (5)	0.3378 (3)	0.0297 (8)	0.54 (2)
H4a1	0.126 (2)	0.672 (2)	0.2865 (16)	0.0446 (13)*	0.54 (2)
H4a2	0.223 (3)	0.559 (3)	0.3363 (18)	0.0446 (13)*	0.54 (2)
H4a3	0.2086 (19)	0.693 (2)	0.4071 (15)	0.0446 (13)*	0.54 (2)
C4b	0.2449 (9)	0.6202 (11)	0.3523 (4)	0.0425 (19)	0.46 (2)
H4b1	0.153 (3)	0.639 (4)	0.320 (3)	0.064 (3)*	0.46 (2)
H4b2	0.272 (3)	0.527 (3)	0.342 (2)	0.064 (3)*	0.46 (2)
H4b3	0.263 (3)	0.626 (3)	0.428 (2)	0.064 (3)*	0.46 (2)
C10d	0.5545 (17)	0.8358 (16)	0.0054 (8)	0.049 (3)	0.39 (3)
H10d1	0.564 (5)	0.751 (5)	−0.028 (4)	0.074 (4)*	0.39 (3)
H10d2	0.477 (4)	0.869 (4)	−0.046 (3)	0.074 (4)*	0.39 (3)
H10d3	0.616 (4)	0.909 (4)	0.006 (3)	0.074 (4)*	0.39 (3)
C10c	0.5817 (7)	0.8001 (10)	0.0173 (6)	0.0452 (14)	0.61 (3)
H10c1	0.606 (2)	0.707 (2)	0.015 (2)	0.068 (2)*	0.61 (3)
H10c2	0.511 (3)	0.823 (3)	−0.050 (2)	0.068 (2)*	0.61 (3)
H10c3	0.660 (2)	0.857 (3)	0.0249 (17)	0.068 (2)*	0.61 (3)

### (E)-3,4,5-Trihydroxy-N'-(3,4,5-trimethoxybenzylidene)benzohydrazide monohydrate Atomic displacement parameters (Å^2^)

**Table:**

	*U*^11^	*U*^22^	*U*^33^	*U*^12^	*U*^13^	*U*^23^
O23	0.0230 (2)	0.0151 (2)	0.0147 (2)	−0.00580 (18)	0.00659 (19)	−0.00383 (17)
O21	0.0317 (3)	0.0189 (2)	0.0142 (2)	−0.0083 (2)	0.0119 (2)	−0.00423 (18)
O17	0.0275 (3)	0.0186 (2)	0.0125 (2)	−0.0064 (2)	0.00826 (19)	−0.00412 (18)
O3	0.0381 (3)	0.0441 (4)	0.0216 (3)	−0.0233 (3)	0.0174 (3)	−0.0159 (3)
O6	0.0155 (2)	0.0193 (2)	0.0202 (2)	−0.00291 (18)	0.00158 (18)	−0.00466 (19)
O9	0.0320 (3)	0.0398 (3)	0.0267 (3)	−0.0219 (3)	0.0137 (2)	−0.0063 (3)
O25	0.0248 (3)	0.0230 (3)	0.0232 (3)	−0.0101 (2)	0.0143 (2)	−0.0044 (2)
N15	0.0242 (3)	0.0144 (2)	0.0106 (2)	−0.0058 (2)	0.0032 (2)	−0.00096 (19)
N14	0.0210 (3)	0.0133 (2)	0.0111 (2)	−0.0041 (2)	0.0024 (2)	−0.00061 (19)
C22	0.0155 (3)	0.0123 (3)	0.0113 (3)	−0.0021 (2)	0.0047 (2)	−0.0009 (2)
C20	0.0185 (3)	0.0133 (3)	0.0113 (3)	−0.0037 (2)	0.0058 (2)	−0.0017 (2)
C19	0.0212 (3)	0.0159 (3)	0.0130 (3)	−0.0069 (2)	0.0077 (2)	−0.0030 (2)
C18	0.0170 (3)	0.0131 (3)	0.0112 (3)	−0.0028 (2)	0.0047 (2)	−0.0023 (2)
C16	0.0183 (3)	0.0131 (3)	0.0106 (3)	−0.0024 (2)	0.0039 (2)	−0.0017 (2)
C13	0.0218 (3)	0.0150 (3)	0.0119 (3)	−0.0044 (2)	0.0027 (2)	−0.0005 (2)
C12	0.0174 (3)	0.0137 (3)	0.0125 (3)	−0.0032 (2)	0.0030 (2)	0.0001 (2)
C1	0.0182 (3)	0.0173 (3)	0.0153 (3)	−0.0059 (2)	0.0060 (2)	−0.0030 (2)
C2	0.0189 (3)	0.0195 (3)	0.0146 (3)	−0.0064 (2)	0.0063 (2)	−0.0047 (2)
C5	0.0149 (3)	0.0153 (3)	0.0155 (3)	−0.0030 (2)	0.0033 (2)	−0.0019 (2)
C7	0.0271 (4)	0.0226 (4)	0.0480 (6)	−0.0002 (3)	−0.0028 (4)	−0.0147 (4)
C8	0.0184 (3)	0.0196 (3)	0.0160 (3)	−0.0067 (2)	0.0052 (2)	−0.0003 (2)
C11	0.0223 (3)	0.0198 (3)	0.0131 (3)	−0.0071 (2)	0.0052 (2)	−0.0002 (2)
C26	0.0188 (3)	0.0159 (3)	0.0135 (3)	−0.0038 (2)	0.0079 (2)	−0.0036 (2)
C24	0.0159 (3)	0.0145 (3)	0.0136 (3)	−0.0033 (2)	0.0066 (2)	−0.0021 (2)
O27	0.0203 (2)	0.0206 (3)	0.0272 (3)	−0.0067 (2)	−0.0013 (2)	0.0062 (2)
C4a	0.0357 (17)	0.0344 (16)	0.0244 (12)	−0.0135 (12)	0.0179 (12)	−0.0065 (10)
C4b	0.050 (3)	0.060 (4)	0.0238 (15)	−0.032 (3)	0.0208 (17)	−0.0121 (19)
C10d	0.059 (6)	0.060 (5)	0.036 (3)	−0.038 (4)	0.028 (4)	−0.011 (3)
C10c	0.043 (2)	0.065 (4)	0.0345 (16)	−0.0290 (19)	0.0243 (15)	−0.0085 (18)

### (E)-3,4,5-Trihydroxy-N'-(3,4,5-trimethoxybenzylidene)benzohydrazide monohydrate Geometric parameters (Å, º)

**Table:**

O23—H231	0.940 (10)	C12—C1	1.3962 (9)
O23—C22	1.3629 (8)	C12—C11	1.3960 (9)
O21—H211	0.955 (11)	C1—H11	1.070 (8)
O21—C20	1.3603 (8)	C1—C2	1.3894 (9)
O17—C16	1.2356 (8)	C2—C5	1.4004 (9)
O3—C2	1.3499 (8)	C5—C8	1.3985 (9)
O3—C4a	1.420 (3)	C7—H71	1.072 (11)
O3—C4b	1.471 (6)	C7—H72	1.070 (11)
O6—C5	1.3729 (8)	C7—H73	1.057 (11)
O6—C7	1.4295 (10)	C8—C11	1.3979 (9)
O9—C8	1.3523 (8)	C11—H111	1.075 (8)
O9—C10d	1.421 (9)	C26—H261	1.075 (8)
O9—C10c	1.436 (6)	C26—C24	1.3875 (9)
O25—H251	0.954 (11)	O27—H271	0.937 (11)
O25—C24	1.3587 (8)	O27—H272	0.973 (12)
N15—H151	0.985 (9)	C4a—H4a1	1.09 (2)
N15—N14	1.3656 (7)	C4a—H4a2	1.10 (3)
N15—C16	1.3596 (8)	C4a—H4a3	1.07 (2)
N14—C13	1.2835 (8)	C4b—H4b1	1.07 (3)
C22—C20	1.4041 (9)	C4b—H4b2	1.09 (3)
C22—C24	1.4006 (9)	C4b—H4b3	1.07 (3)
C20—C19	1.3899 (9)	C10d—H10d1	1.06 (5)
C19—H191	1.065 (8)	C10d—H10d2	1.09 (4)
C19—C18	1.3996 (9)	C10d—H10d3	1.07 (4)
C18—C16	1.4844 (9)	C10c—H10c1	1.05 (2)
C18—C26	1.3903 (9)	C10c—H10c2	1.14 (3)
C13—H131	1.071 (8)	C10c—H10c3	1.08 (3)
C13—C12	1.4637 (9)		
			
C22—O23—H231	113.2 (6)	H71—C7—O6	108.0 (6)
C20—O21—H211	114.7 (6)	H72—C7—O6	109.8 (6)
C4a—O3—C2	116.66 (17)	H72—C7—H71	112.2 (8)
C4b—O3—C2	115.9 (2)	H73—C7—O6	106.7 (6)
C4b—O3—C4a	23.2 (3)	H73—C7—H71	108.3 (8)
C7—O6—C5	113.86 (6)	H73—C7—H72	111.5 (8)
C10d—O9—C8	118.1 (5)	C5—C8—O9	115.08 (6)
C10c—O9—C8	116.9 (3)	C11—C8—O9	125.18 (6)
C10c—O9—C10d	20.3 (6)	C11—C8—C5	119.74 (6)
C24—O25—H251	112.3 (6)	C8—C11—C12	119.24 (6)
N14—N15—H151	119.0 (5)	H111—C11—C12	119.3 (4)
C16—N15—H151	122.4 (5)	H111—C11—C8	121.4 (4)
C16—N15—N14	118.06 (5)	H261—C26—C18	119.8 (4)
C13—N14—N15	117.33 (5)	C24—C26—C18	121.00 (6)
C20—C22—O23	117.09 (5)	C24—C26—H261	119.2 (4)
C24—C22—O23	123.53 (6)	C22—C24—O25	122.85 (6)
C24—C22—C20	119.36 (6)	C26—C24—O25	117.49 (6)
C22—C20—O21	116.03 (5)	C26—C24—C22	119.66 (6)
C19—C20—O21	123.28 (6)	H272—O27—H271	112.1 (9)
C19—C20—C22	120.64 (6)	H4a1—C4a—O3	116.6 (12)
H191—C19—C20	117.5 (4)	H4a2—C4a—O3	108.7 (13)
C18—C19—C20	119.59 (6)	H4a2—C4a—H4a1	102 (2)
C18—C19—H191	122.8 (4)	H4a3—C4a—O3	104.8 (11)
C16—C18—C19	123.54 (6)	H4a3—C4a—H4a1	111.8 (17)
C26—C18—C19	119.74 (6)	H4a3—C4a—H4a2	112.8 (18)
C26—C18—C16	116.63 (5)	H4b1—C4b—O3	105 (2)
N15—C16—O17	121.72 (6)	H4b2—C4b—O3	115.9 (17)
C18—C16—O17	121.73 (6)	H4b2—C4b—H4b1	114 (3)
C18—C16—N15	116.53 (5)	H4b3—C4b—O3	109.0 (15)
H131—C13—N14	121.5 (4)	H4b3—C4b—H4b1	112 (2)
C12—C13—N14	119.45 (6)	H4b3—C4b—H4b2	101 (2)
C12—C13—H131	119.1 (4)	H10d1—C10d—O9	116 (3)
C1—C12—C13	119.61 (6)	H10d2—C10d—O9	112 (2)
C11—C12—C13	119.04 (6)	H10d2—C10d—H10d1	98 (4)
C11—C12—C1	121.32 (6)	H10d3—C10d—O9	107 (2)
H11—C1—C12	119.6 (4)	H10d3—C10d—H10d1	121 (3)
C2—C1—C12	119.18 (6)	H10d3—C10d—H10d2	101 (3)
C2—C1—H11	121.2 (4)	H10c1—C10c—O9	112.6 (13)
C1—C2—O3	124.42 (6)	H10c2—C10c—O9	108.9 (16)
C5—C2—O3	115.43 (6)	H10c2—C10c—H10c1	109 (2)
C5—C2—C1	120.15 (6)	H10c3—C10c—O9	105.0 (13)
C2—C5—O6	119.95 (6)	H10c3—C10c—H10c1	109.4 (18)
C8—C5—O6	119.65 (6)	H10c3—C10c—H10c2	112.3 (19)
C8—C5—C2	120.35 (6)		
			
O23—C22—C20—O21	−3.99 (7)	O25—C24—C26—C18	−178.76 (6)
O23—C22—C20—C19	178.55 (6)	N15—N14—C13—C12	−178.94 (6)
O23—C22—C24—O25	0.70 (8)	N15—C16—C18—C19	12.19 (7)
O23—C22—C24—C26	−178.59 (6)	N15—C16—C18—C26	−171.26 (6)
O21—C20—C22—C24	177.39 (6)	N14—C13—C12—C1	−17.48 (8)
O21—C20—C19—C18	−177.57 (7)	N14—C13—C12—C11	160.80 (7)
O17—C16—N15—N14	4.59 (8)	C22—C20—C19—C18	−0.30 (8)
O17—C16—C18—C19	−166.24 (7)	C22—C24—C26—C18	0.57 (8)
O17—C16—C18—C26	10.31 (8)	C20—C19—C18—C16	177.24 (6)
O3—C2—C1—C12	−179.60 (7)	C20—C19—C18—C26	0.80 (8)
O3—C2—C5—O6	3.49 (8)	C19—C18—C26—C24	−0.94 (8)
O3—C2—C5—C8	−179.20 (6)	C13—C12—C1—C2	176.87 (6)
O6—C5—C2—C1	−175.92 (6)	C13—C12—C11—C8	−176.43 (6)
O6—C5—C8—O9	−2.67 (7)	C12—C1—C2—C5	−0.25 (8)
O6—C5—C8—C11	176.38 (6)	C12—C11—C8—C5	−0.65 (8)
O9—C8—C5—C2	−179.99 (6)	C1—C2—C5—C8	1.40 (8)
O9—C8—C11—C12	178.30 (7)	C2—C5—C8—C11	−0.94 (8)
O25—C24—C22—C20	179.23 (6)		

### (E)-3,4,5-Trihydroxy-N'-(3,4,5-trimethoxybenzylidene)benzohydrazide monohydrate Hydrogen-bond geometry (Å, º)

**Table:**

*D*—H···*A*	*D*—H	H···*A*	*D*···*A*	*D*—H···*A*
O23—H231···O27^i^	0.941 (10)	1.748 (10)	2.6892 (7)	179.3 (9)
O21—H211···O17^ii^	0.955 (11)	1.790 (11)	2.7246 (7)	165.3 (10)
O25—H251···O27^i^	0.954 (11)	1.811 (11)	2.7649 (8)	177.5 (10)
N15—H151···O23^iii^	0.985 (9)	2.115 (9)	3.0491 (7)	157.7 (8)
N15—H151···O21^iii^	0.985 (9)	2.320 (9)	2.9572 (8)	121.6 (7)
C19—H191···O23^iii^	1.065 (8)	2.363 (8)	3.3661 (8)	156.4 (6)
C11—H111···O3^iv^	1.076 (8)	2.555 (9)	3.3986 (9)	134.7 (6)
O27—H271···O6^v^	0.938 (11)	1.858 (12)	2.7746 (8)	165.1 (10)
O27—H272···N14	0.973 (12)	1.956 (12)	2.8930 (8)	161.0 (10)
C10*d*—H10*d*2···O3^iv^	1.09 (4)	2.59 (5)	3.472 (16)	138 (3)
C10*d*—H10*d*3···O25^vi^	1.07 (4)	2.47 (4)	3.236 (11)	128 (3)
C10*c*—H10*c*3···O25^vi^	1.08 (3)	2.41 (2)	3.229 (7)	131.4 (17)

Symmetry codes: (i) −*x*, −*y*, −*z*; (ii) *x*, −*y*+1/2, *z*−1/2; (iii) −*x*, *y*+1/2, −*z*−1/2; (iv) *x*, −*y*+3/2, *z*−1/2; (v) −*x*+1, *y*−1/2, −*z*+1/2; (vi) *x*+1, *y*+1, *z*.

## References

[bb1] Alhadi, A. A., Saharin, S. M., Mohd Ali, H., Robinson, W. T. & Abdulla, M. A. (2009). *Acta Cryst.* E**65**, o1373. 10.1107/S1600536809018947 PMC2969598 21583222

[bb2] Alhadi, A. A., Shaker, S. A., Suleiman, N., Yehye, W. A. & Mohd Ali, H. (2012). *J. Chil. Chem. Soc.* **57**, 1283–1286.

[bb3] Badhani, B., Sharma, N. & Kakkar, R. (2015). *RSC Adv.* **5**, 27540–27557.

[bb4] Bourhis, L. J., Dolomanov, O. V., Gildea, R. J., Howard, J. A. K. & Puschmann, H. (2015). *Acta Cryst.* A**71**, 59–75. 10.1107/S2053273314022207 PMC4283469 25537389

[bb5] Bruno, I. J., Cole, J. C., Kessler, M., Luo, J., Motherwell, W. D. S., Purkis, L. H., Smith, B. R., Taylor, R., Cooper, R. I., Harris, S. E. & Orpen, A. G. (2004). *J. Chem. Inf. Comput. Sci.* **44**, 2133–2144. 10.1021/ci049780b 15554684

[bb6] Dolomanov, O. V., Bourhis, L. J., Gildea, R. J., Howard, J. A. K. & Puschmann, H. (2009). *J. Appl. Cryst.* **42**, 339–341.

[bb7] Feldblum, E. S. & Arkin, I. (2024). *Proc. Natl. Acad. Sci.* **111**, 4085–4090. 10.1073/pnas.1319827111 PMC3964065 24591597

[bb8] Galek, P. T. A., Chisholm, J. A., Pidcock, E. & Wood, P. A. (2014). *Acta Cryst.* B**70**, 91–105. 10.1107/S2052520613033003 24441132

[bb9] Groom, C. R., Bruno, I. J., Lightfoot, M. P. & Ward, S. C. (2016). *Acta Cryst.* B**72**, 171–179. 10.1107/S2052520616003954 PMC4822653 27048719

[bb10] Hadidi, M., Liñán-Atero, R., Tarahi, M., Christodoulou, M. C. & Aghababaei, F. (2024). *Antioxidants*, **13**, 1001. 10.3390/antiox13081001 PMC11352096 39199245

[bb11] Haranahalli, K., Lazzarini, C., Sun, Y., Zambito, J., Pathiranage, S., McCarthy, J. B., Mallamo, J., Del Poeta, M. & Ojima, I. (2019). *J. Med. Chem.* **62**, 8249–8273. 10.1021/acs.jmedchem.9b01004 PMC6755904 31369263

[bb12] Humphrey, W., Dalke, A. & Schulten, K. (1996). *J. Mol. Graph.* **14**, 1996, 33–38. 10.1016/0263-7855(96)00018-5 8744570

[bb13] Johnson, E. R., Keinan, S., Mori-Sánchez, P., Contreras-García, J., Cohen, A. J. & Yang, W. (2010). *J. Am. Chem. Soc.* **132**, 6498–6506. 10.1021/ja100936w PMC2864795 20394428

[bb14] Kassab, A. E. (2023). *Arch. Pharm.* **356**, 2200548.

[bb15] Kleemiss, F., Dolomanov, O. V., Bodensteiner, M., Peyerimhoff, N., Midgley, L., Bourhis, L. J., Genoni, A., Malaspina, L. A., Jayatilaka, D., Spencer, J. L., White, F., Grundkötter-Stock, B., Steinhauer, S., Lentz, D., Puschmann, H. & Grabowsky, S. (2021). *Chem. Sci.* **12**, 1675–1692. 10.1039/d0sc05526c PMC8179328 34163928

[bb16] Krause, L., Herbst-Irmer, R., Sheldrick, G. M. & Stalke, D. (2015). *J. Appl. Cryst.* **48**, 3–10. 10.1107/S1600576714022985 PMC4453166 26089746

[bb17] Liu, R., Cui, J., Ding, T., Liu, Y. & Liang, H. (2022). *Molecules* **27**, 8393. 10.3390/molecules27238393 PMC9739244 36500482

[bb18] Liu, Y., Peng, Q., Li, Y., Hou, H. & Li, K. (2020). *Chin. Chem. Lett.* **31**, 3271–3275.

[bb19] Macrae, C. F., Sovago, I., Cottrell, S. J., Galek, P. T. A., McCabe, P., Pidcock, E., Platings, M., Shields, G. P., Stevens, J. S., Towler, M. & Wood, P. A. (2020). *J. Appl. Cryst.* **53**, 226–235. 10.1107/S1600576719014092 PMC6998782 32047413

[bb20] Maia, R., Tesch, R. & Fraga, C. A. M. (2014). *Expert Opin. Ther. Pat.* **24**, 1161–1170. 10.1517/13543776.2014.959491 25213630

[bb21] Momany, F. A. & Rone, R. (1992). *J. Comput. Chem.* **13**, 888–900.

[bb22] Neese, F. (2012). *WIREs Comput. Mol. Sci.* **2**, 73–78.

[bb23] Oliveira, F. A., Pinto, A. C. S., Duarte, C. L., Taranto, A. G., Lorenzato Junior, E., Cordeiro, C. F., Carvalho, D. T., Varotti, F. P. & Fonseca, A. L. (2022). *BMC Chem.* **16**, 50. 10.1186/s13065-022-00843-9 PMC9271247 35810303

[bb24] Palatinus, L. & Chapuis, G. (2007). *J. Appl. Cryst.* **40**, 786–790.

[bb25] Peng, Z., Li, Y., Tan, L., Chen, L., Shi, Q., Zeng, Q.-H., Liu, H., Wang, J. J. & Zhao, Y. (2022). *Food Chem.* **378**, 132127. 10.1016/j.foodchem.2022.132127 35033723

[bb26] Ramimoghadam, D., Eyckens, D. J., Evans, R. A., Moad, G., Holmes, S. & Simons, R. (2024). *Chem. A Eur. J.* **30**, e202401728. 10.1002/chem.202401728 38888459

[bb27] Shaikh, A., Ghosh, M., Mukherjee, P., Ghosh, A., Molla, R. A., Ta, S. & Das, D. (2020). *New J. Chem.* **44**, 13501–13506.

[bb28] Socea, L.-I., Barbuceanu, S.-F., Pahontu, E. M., Dumitru, A.-C., Nitulescu, G. M., Sfetea, R. C. & Apostol, T.-V. (2022). *Molecules*, **27**, 8719. 10.3390/molecules27248719 PMC9783609 36557851

[bb29] van Dijken, D. J., Kovaříček, P., Ihrig, S. P. & Hecht, S. (2015). *J. Am. Chem. Soc.* **137**, 14982–14991. 10.1021/jacs.5b09519 26580808

[bb30] Vlad, I. M., Nuţă, D. C., Căproiu, M. T., Dumitraşcu, F., Kapronczai, E., Mük, G. R., Avram, S., Niculescu, A. G., Zarafu, I., Ciorobescu, V. A., Brezeanu, A. M. & Limban, C. (2024). *Antibiotics*, **13**, 212. 10.3390/antibiotics13030212 PMC10967372 38534647

[bb31] Zhou, X., Zeng, L., Chen, Y., Wang, X., Liao, Y., Xiao, Y., Fu, X. & Yang, Z. (2020). *Int. J. Mol. Sci.* **21**, 5684. 10.3390/ijms21165684 PMC7460824 32784431

